# A trans-Atlantic perspective on successful plantation establishment in boreal ecosystems: lessons learned and research opportunities

**DOI:** 10.1007/s11056-024-10086-2

**Published:** 2024-12-04

**Authors:** Nelson Thiffault, Per Nordin, Amy Wotherspoon, Karin Hjelm, Erika Olofsson

**Affiliations:** 1 Natural Resources Canada, Canadian Forest Service, Québec, QC Canada; 2 Centre d’étude de la forêt, Montréal, QC Canada; 3 pcSKOG AB, Lund, Sweden; 4 University of British Columbia, Vancouver, BC Canada; 5 Swedish University of Agricultural Sciences, Southern Swedish Forest Research Centre, Alnarp, Sweden; 6 Department of Forestry and Wood Technology, Linnaeus University, Växjö, Sweden

**Keywords:** Boreal forests, Reforestation, Regeneration, Climate change adaptation, Mechanical Site Preparation (MSP), Silviculture

## Abstract

Boreal forests, which account for one-third of the world’s forested areas, play a crucial role in global climate regulation and provide significant ecological, economic, and cultural benefits. However, boreal ecosystems face substantial threats from climate change, leading to increased disturbances such as wildfires, insect outbreaks, and disease. In response, reforestation emerges as a vital strategy for maintaining and restoring forest cover. In this perspective paper, we summarize some recent research on plantation establishment in boreal ecosystems of eastern North America and Scandinavia, emphasizing the effectiveness of mechanical site preparation (MSP), species-specific responses, and soil nutrient dynamics. We suggest key areas for future research, including the long-term sustainability of MSP, the development of adaptive strategies to climate variability, species-specific optimization of planting techniques, and integration of technological advances. Addressing these research needs will support the development of adaptive silviculture practices that enhance boreal stands resilience and productivity, helping to meet reforestation objectives and mitigate the impacts of climate change. We aim to stimulate regional, national, and international research initiatives, contributing to the resilience and sustainability of boreal ecosystems.

## Introduction

Boreal forests account for approximately one-third of the world’s forests. They are crucial for global climate regulation through their role in energy, water and gas exchange. Boreal forests are significant carbon reservoirs: estimates suggest that the total carbon reserve in the circumboreal zone ranges from 272 to 1715 billion tons (Bradshaw and Warkentin 2015; Pan et al. 2011). Beyond their ecological value, boreal forests have substantial economic significance, providing timber and pulp resources, biomass for bioenergy, and supporting recreational and ecotourism activities (Börjesson et al. 2017; Gauthier et al. 2015; Paré et al. 2016). Communities rely on them for fishing, hunting, gathering, recreation, and economic activities (Burton et al. 2010). In addition, they are central to the cultural, spiritual and medicinal traditions of many Indigenous communities (Bélisle and Asselin 2021). Finally, boreal forests support a wide range of plant and animal species, contributing to global biodiversity and offering wildlife habitats (Martin et al. 2023).

**Fig. 1:**
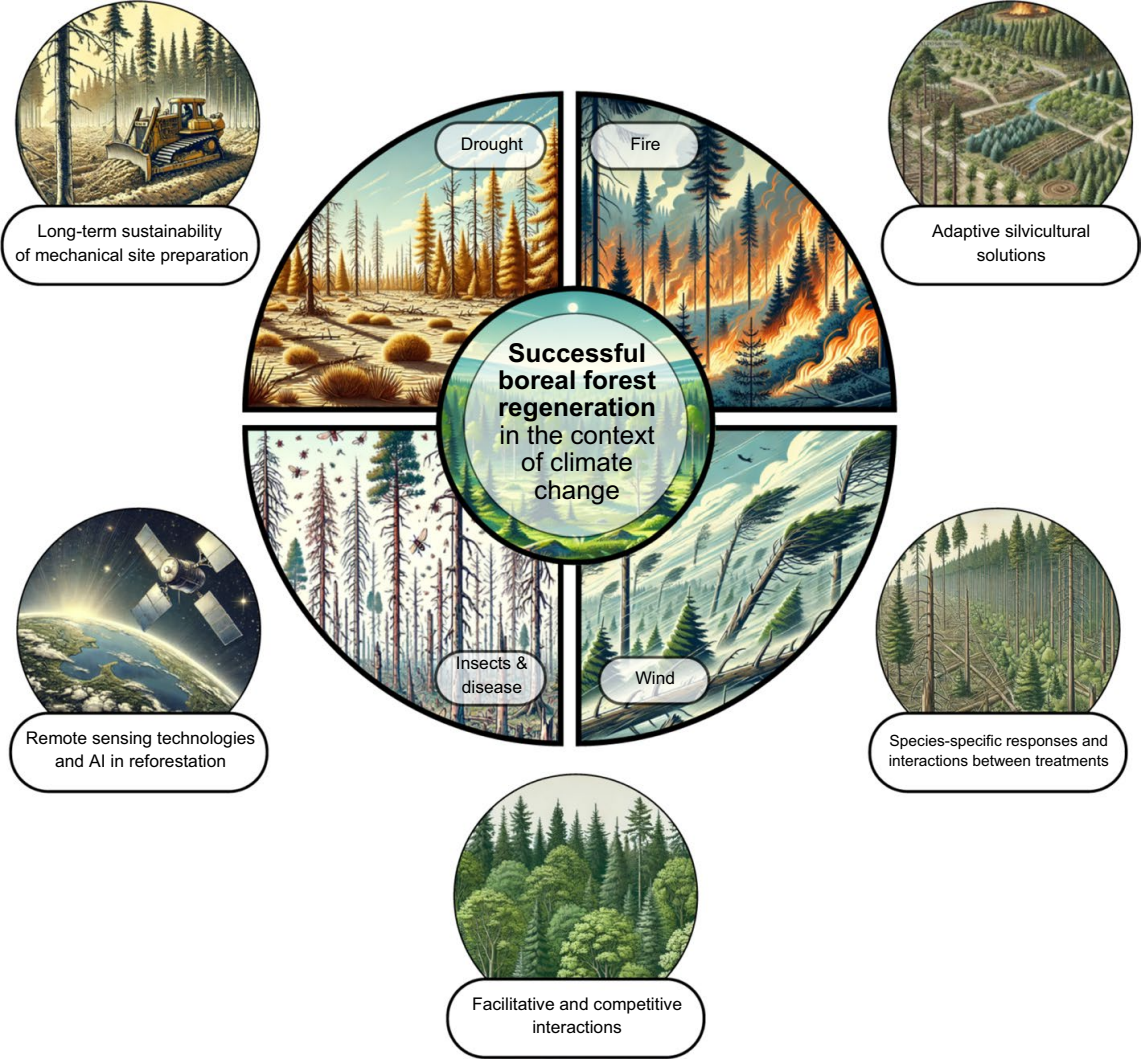
Five components of research that address the four predominant stressors to boreal forests due to climate change in order to ensure successful regeneration

Global change poses a threat to boreal forests, with its impacts expected to be more pronounced in these regions than elsewhere (Gauthier et al. 2015; Wotherspoon et al. 2024). Projected changes in temperature and moisture may have an overall beneficial but limited effect on forest regrowth rates (Danneyrolles et al. 2023; Wang et al. 2023), but these effects might be transitory (D’Orangeville et al. 2018). Moreover, there is a substantial risk of permanent loss of boreal forest cover as climate change affects the survival, establishment, and growth of boreal tree species. Regeneration failures are likely to become more frequent following natural disturbances such as wildfires (Boucher et al. 2020), whose risks are exacerbated by climate change (Ellis et al. 2022). For instance, in Québec, eastern Canada, the 2023 wildfire season set records due to extreme warm and dry conditions, burning 4.5 million ha; this level of wildfire activity significantly affects forest productivity, timber supply, and the socio-economic stability of forest-dependent communities (Boulanger et al. 2024). Drought and heat are key drivers of increased forest mortality (Senf et al. 2020) and substantial reductions in forest growth and carbon sequestration (Laudon et al. 2024). Moreover, wind storms, insect outbreaks and disease risks are increasing with rising temperatures, further threatening forest stability and regeneration (Blennow et al. 2010; D’Orangeville et al. 2023; Gardiner et al. 2013; Hlásny et al. 2021; Venäläinen et al. 2020). The cumulative impacts of these factors (Fig. 1), combined with widespread interactions between agents are likely to amplify disturbances (Seidl et al. 2017), and lead to shifts in boreal forest ecosystems towards new, potentially less desirable states.

In response to these challenges, reforestation is emerging as a key strategy to maintain or restore forest cover following both natural and anthropogenic disturbances (Cyr et al. 2022). It is also a crucial tool for adapting forests to climate change, particularly through breeding, selection and assisted migration of tree species (Bolte et al. 2009; Keskitalo et al. 2016; Palik et al. 2022). While it is not beneficial in all contexts (Kirschbaum et al. 2023), tree planting is widely regarded as a natural climate solution to mitigate the impacts of climate change (Bastin et al. 2019; Drever et al. 2021). Some countries within the circumboreal region have committed to large-scale tree planting initiatives, such as Canada’s 2 Billion Trees program, or the EU’s biodiversity strategy for 2030 that includes the planting of 3 billion trees. These initiatives, for instance, aim to capture carbon, enhance biodiversity, improve forests and societies’ resilience to climate change, support human well-being, and reduce the risk of wildland fires and floods to communities.

However, economic incentives and a greater focus on the boreal biome in international forums are needed to support these adaptation and mitigation actions (Gauthier et al. 2015). Indeed, the establishment of planted seedlings in boreal environments faces significant challenges (Grossnickle 2000). Factors such as relatively poor soils, short growing seasons, temperature extremes, insect damage, and competition from early successional species can limit seedling survival and early growth (Luoranen et al. 2023). These challenges, coupled with planted species fitness and resilience to climate change (Robert et al. 2024), can undermine the capacity of tree planting programs to achieve their objectives. The establishment of plantations in boreal ecosystems has been a significant research topic in recent decades, especially in North America and Scandinavia. However, global change brings new complexities to this field. Increased public scrutiny of forestry operations, the need for reconciliation with Indigenous Peoples, and the rising risk of invasions by non-native plant and insect species due to warmer climates are all factors that must be considered.

Boreal forests are characterized by harsh climatic conditions and unique ecological dynamics. They present both challenges and opportunities for sustainable plantation practices. Recent studies have provided valuable insights into various aspects of plantation establishment, including mechanical site preparation (MSP), species-specific responses, soil nutrient dynamics, and the impact of climate variability. Our objectives in this perspective paper are to summarize some of the recent research on plantation establishment in boreal ecosystems of eastern North America and Scandinavia, and to identify key areas for future research initiatives. We did not intend to conduct a systematic literature review. Instead, based on our experience, we aimed to highlight key recent developments and research needs in the field, to stimulate regional, national, and international research efforts in boreal reforestation and restoration. By doing so, we hope to support the development of adaptive silviculture practices to climate change and help meet the reforestation objectives of northern countries.

## Plantation establishment in boreal ecosystems: recent research and future directions

One of the primary focus areas in recent research has been the effects of MSP on soil health and tree growth. Several studies have highlighted that MSP in boreal ecosystems offers short-and mid-term benefits by creating suitable planting microsites and reducing competition by shrub species. For example, there are sustained growth benefits to planted conifers many years post-MSP (Wotherspoon et al. 2020), yet there is a potential for these effects to diminish over time due to changing competitive dynamics with the shrub layer (Reicis et al. 2023). Boreal forest soil biotic communities are also affected by site preparation (Peck et al. 2016; Smenderovac et al. 2023), which can impact both tree growth and soil C stocks. There is a need for better understanding of carbon stock changes due to MSP (Dufour et al. 2024; Mäkipää et al. 2023; Mjöfors et al. 2017), which can vary significantly based on climatic and microsite conditions. While short (Nilsson et al. 2019), mid- (Uotila et al. 2022) and longer term (Hjelm et al. 2019) studies have reported positive growth responses of planted species to MSP, there is a need for long-term studies to understand the persistence of these effects on nutrient availability and soil health (Ring and Sikström 2024). Recent results further highlight the importance of nutrient management and soil health in optimizing plantation outcomes (Nilsson et al. 2024). There is, overall, a need to investigate the long-term sustainability of MSP by assessing its effects on soil nutrient dynamics, microbial community dynamics, and forest productivity over several decades (Sutinen et al. 2010, 2019). This research should include comprehensive longitudinal studies that monitor changes in soil properties and nutrient profiles to ensure the benefits of MSP are maintained without compromising ecosystem health.

Research has highlighted the impact of climate variability on MSP outcomes. Climate conditions, such as cooler and wetter regimes, significantly influence the effectiveness of MSP (Henneb et al. 2020). Studies on this topic have underlined the importance of understanding how different climatic conditions affect the long-term success of MSP treatments and tree growth (Sikström et al. 2020), particularly under the anticipated effects of climate change. Given the projected shifts in climate patterns, it is crucial to develop adaptive silviculture strategies that can maintain their efficacy across diverse climatic scenarios (Achim et al. 2022). MSP has shown the potential to increase water use efficiency in some species (Wotherspoon et al. 2020), but more research is required to identify resilient species and genotypes, and develop strategies to mitigate the impacts of biotic and abiotic stressors. Research should focus on enhancing seedling growth and survival under various stress conditions, investigating genetic and physiological responses of tree species to climate and different silvicultural practices (Robert et al. 2024), and aiming to identify best practices for different species and site conditions to enhance resilience and productivity through genetic adaptation and phenotypic plasticity.

Forest assisted migration is a central component of many adaptive silviculture strategies (e.g., Nagel et al. 2017). This approach involves relocating tree species or genotypes from their native climates to areas projected to have similar conditions in the future, with the aim of preserving and sustaining stand function, productivity, and overall ecosystem health (Pedlar et al. 2012). When implemented effectively, assisted migration of tree species is considered a sine qua non for preserving the forest carbon sink under climate change (Chakraborty et al. 2024; Pedlar 2024). In Canada and other regions, the movement of seed sources within existing species’ range limits is already being implemented for a few commercial species, guided by climatic matching of seedlots (McKenney et al. 1999; St.Clair et al. 2022). Large-scale silviculture experiments are being established to examine the interactions between silvicultural systems and forest assisted migration (e.g., Royo et al. 2023; Thiffault et al. 2024). While short-term results related to regeneration growth and survival are expected soon, mid- and long-term findings from these studies will be essential for informing deployment practices and shaping policy.

Species-specific responses to MSP and planting techniques have also been a subject of extensive research. Most research on MSP and plant performance has been conducted using few conifer tree species (Löf et al. 2012). Tree species exhibit varied responses to MSP (Nordin et al. 2023; Thiffault et al. 2010), illustrating that one-size-fits-all approaches are not optimal. This variability requires further research to optimize MSP and planting techniques tailored to specific species to maximize growth and survival rates. Additionally, there is a need to further explore the interactions between MSP and other silvicultural treatments such as fertilization (Thiffault and Jobidon 2006) and stock types (Johansson et al. 2007; Thiffault et al. 2012); research efforts should address silvicultural strategies that consider these complex interactions to enhance reforestation success, such as those between planting position, seedling size, and organic fertilizers (Häggström et al. 2021, 2024). This includes understanding how different species respond to reforestation practices under a range of environmental conditions to maximize growth and survival rates (Luoranen et al. 2023, 2024), as well as further our understanding of the interactions between drought, insect damages, and protection measures (Domevscik et al. 2024; Wallertz et al. 2024).

The role of facilitation and competition interactions in seedling establishment also requires further investigation. For instance, some species like alder (*Alnus* spp.) can play a dual role in forest renewal, as they can both facilitate and compete with target tree species (Urli et al. 2020). Climate shifts will lead to changes in understory plant communities (Chalumeau et al. 2024; Villén‐Peréz et al. 2020), potentially introducing new facilitative and competitive interactions for boreal tree species. With warmer conditions, neighboring vegetation is expected to develop more rapidly, increasing competition with seedlings. This stresses the need for ongoing research to adapt vegetation management strategies, including potentially intensifying or increasing the frequency of mechanical site preparation (MSP) and other control methods, to ensure successful forest regeneration (Thiffault 2021). Understanding the interactions between silviculture and potential nurse species (Thiffault and Hébert 2017), as well as between planted species (Roy Proulx et al. 2024a) is vital for developing management practices that balance these effects while taking advantage of mixed plantations (Löf et al. 2014). There is an overall need to investigate the roles of different species in mixed stands, focusing on their facilitative and competitive interactions (e.g., Roy Proulx et al. 2024b), to promote the best species mixtures in terms of survival and growth when establishing plantations (Aldea et al. 2024). Moreover, we note the need to pursue research efforts on the restoration of mixed stands from pure plantations (Löf et al. 2023), to favor more diverse and resilient forest types in the face of global change. While the restoration of mixed stands is a priority, the low number of tree species traditionally used in boreal forestry, such as in Scandinavia and Canada, presents challenges. These constraints necessitate region-specific research approaches to identify suitable species combinations and management practices that promote diversity and resilience.

Remote sensing technologies significantly influence forest management and silviculture by enabling the observation and mapping of forest stand composition, understory vegetation, and soil properties using ground-based, aerial, and satellite platforms (Almeida et al. 2019; White et al. 2021). Advancements in drone system imagery and computer vision, for example, now facilitate the automatic identification and counting of planting microsites, making reforestation efforts more precise and efficient (Bouachir et al. 2019; Genest et al. 2024). Drone-based photogrammetry, leveraging advanced imaging and automated single stem detection algorithms, has demonstrated promising potential for assessing regeneration performance by evaluating stocking, spatial density, and height distribution of both naturally regenerating and planted conifer stands (e.g., Vepakomma et al. 2015). Further work is needed, however, to improve vegetation differentiation and classification (Goodbody et al. 2017) and enabling use in different stand conditions.

Integration of artificial intelligence (AI) with remote sensing tools also holds great potential to enhance reforestation and forest management efforts (Buchelt et al. 2024). AI-powered drones equipped with advanced imaging technologies can perform tasks such as species identification, canopy height monitoring, and health assessments at unprecedented scales and resolutions. Models are being developed to improve transparency and trust, enabling drones to perform real-time monitoring of reforestation success and even automate corrective actions. Drone systems are also being tested for seed-dispensing operations, combining AI with mission-planning algorithms to autonomously scout and depose seed in optimal locations, particularly in challenging terrains (Siedler 2022).

Multi-sensor drone platforms are changing forest inventory and monitoring, but more work is needed to fully integrate these tools into silvicultural prescriptions (Goodbody et al. 2024). These technological innovations collectively have the potential to improve the efficiency and accuracy of forest management while reducing risks and costs associated with traditional field surveys, supporting the rapid scale-up of mechanized reforestation activities (Ersson et al. 2022; Li et al. 2024; Manner and Ersson 2021; Ramantswana et al. 2020). In that regard, advancements in mechanized planting systems are being pioneered, where automation efforts aim to develop autonomous machines capable of site preparation and tree planting with high precision and low environmental impact (Hansson et al. 2024). Research into mechanized planting highlights the potential to increase the efficiency and quality of tree establishment while addressing challenges like labor shortages and cost efficiency (Ersson et al. 2018).

## Conclusion

Boreal forests are of critical ecological, economic, and cultural importance. As climate change intensifies, the challenges to maintaining and restoring boreal forests will increase. With this perspective paper, we aim to contribute to this ongoing effort by highlighting recent research and proposing research needs for the field (Table 1). The establishment of plantations in boreal ecosystems requires an understanding of various ecological, climatic, and operational factors, including site preparation methods, planting material, and monitoring of reforestation success, all of which are integral to forest management and silviculture treatments (Fig. 1). Recent research has provided valuable insights into these aspects, yet significant gaps remain. Long-term studies on the sustainability of MSP benefits, the development of adaptive strategies to climate variability, species-specific optimization of planting techniques, and the full integration of recent technological advances into silvicultural practices are essential for advancing boreal reforestation efforts. Although not explored in this perspective paper, integrating Indigenous Knowledge into forest renewal research is essential for enhancing the sustainability and resilience of boreal forests. Indigenous perspectives provide invaluable insights into environmental changes and forest management practices, drawn from generations of close connection with the forest (Bélisle et al. 2022). Traditional knowledge is particularly valuable in contexts where field experiments require extended periods to yield results, offering complementary insights that can accelerate understanding and decision-making (Di Sacco et al. 2021; Wangpakapattanawong et al. 2010). However, examples of collaborative forestry research that effectively integrate Indigenous and scientific knowledge systems remain limited (Palaschuk and Bullock 2019). By addressing the identified research needs, we hope to stimulate regional, national, and international research efforts in boreal reforestation and restoration. This should support the development of adaptive silviculture practices to climate change and help meet the reforestation objectives of northern countries.

**Table 1:** Some key research needs in boreal plantation establishment

Research need	Description	Supporting references
Long-term Sustainability of Mechanical Site Preparation	Investigate the long-term sustainability of mechanical site preparation by assessing its effects on soil nutrient dynamics, microbial community dynamics, and forest productivity over several decades	Dufour et al. (2024); Hjelm et al. (2019); Mäkipää et al. (2023); Mjöfors et al. (2017); Nilsson et al. (2019); Nilsson et al. (2024); Peck et al. (2016); Reicis et al. (2023); Ring and Sikström (2024); Smenderovac et al. (2023); Sutinen et al. (2010); Sutinen et al. (2019); Uotila et al. (2022); Wotherspoon et al. (2020)
Adaptive Silvicultural Solutions	Develop adaptive plantation establishment strategies by studying how different climatic conditions affect the success of mechanical site preparation and other silvicultural practices, and identify resilient species and genotypes to develop strategies to mitigate the impacts of biotic and abiotic stressors, including the use of forest assisted migration	Achim et al. (2022); Chakraborty et al. (2024); Henneb et al. (2020); Nagel et al. (2017); Pedlar (2024); Robert et al. (2024); Royo et al. (2023); Sikström et al. (2020; Thiffault et al. (2024)
Species-Specific Responses to Silviculture and Interactions Between Silvicultural Treatments	Optimize mechanical site preparation and planting techniques for various tree species by conducting species-specific research to maximize growth and survival rates and explore the interactions between mechanical site preparation, fertilization, and other silvicultural treatments	Domevscik et al. (2024); Häggström et al. (2021); Häggström et al. (2024); Johansson et al. (2007); Löf et al. (2012); Luoranen et al. (2023); Luoranen et al. (2024); Nordin et al. (2023); Thiffault and Jobidon (2006); Thiffault et al. (2010); Thiffault et al. (2012); Wallertz et al. (2024)
Facilitative and Competitive Interactions	Investigate the roles of different species in mixed stands, focusing on their facilitative and competitive interactions to promote optimal species mixtures under climate change	Aldea et al. (2024); Chalumeau et al. (2024); Löf et al. (2014); Löf et al. (2023); Roy Proulx et al. (2024a); Roy Proulx et al. (2024b); Thiffault (2021); Thiffault and Hébert (2017); Urli et al. (2020); Villén‐Peréz et al. (2020)
Remote Sensing Teachnologies and AI in Reforestation	Integrate remote sensing tools and artificial intelligence to characterize forest landscapes and enhance the effectiveness of mechanized reforestation activities	Almeida et al. (2019); Bouachir et al. (2019); Buchelt et al. (2024); Ersson et al. (2018); Ersson et al. (2022); Genest et al. (2024); Goodbody et al. (2017); Goodbody et al. (2024); Hansson et al. (2024); Li et al. (2024); Manner and Ersson (2021); Ramantswana et al. (2020); Siedler (2022); Vepakomma et al. (2015); White et al. (2021)

## Data Availability

No data was generated during the preparation of this manuscript.
